# Independent validation of the PREDICT breast cancer prognosis prediction tool in 45,789 patients using Scottish Cancer Registry data

**DOI:** 10.1038/s41416-018-0256-x

**Published:** 2018-09-17

**Authors:** Ewan Gray, Joachim Marti, David H. Brewster, Jeremy C. Wyatt, Peter S. Hall, the SATURNE Advisory Group

**Affiliations:** 1 University of Edinburgh, Edinburgh, UK; 2 University of Lausanne, Lausanne, Switzerland; 3 University of Southampton, Southampton, UK

## Abstract

**Background:**

PREDICT is a widely used online prognostication and treatment benefit tool for patients with early stage breast cancer. The aim of this study was to conduct an independent validation exercise of the most up-to-date version of the PREDICT algorithm (version 2) using real-world outcomes from the Scottish population of women with breast cancer.

**Methods:**

Patient data were obtained for all Scottish Cancer Registry (SCR) records with a diagnosis of primary invasive breast cancer diagnosed in the period between January 2001 and December 2015. Prognostic scores were calculated using the PREDICT version 2 algorithm. External validity was assessed by statistical analysis of discrimination and calibration. Discrimination was assessed by area under the receiver-operator curve (AUC). Calibration was assessed by comparing the predicted number of deaths to the observed number of deaths across relevant sub-groups.

**Results:**

A total of 45,789 eligible cases were selected from 61,437 individual records. AUC statistics ranged from 0.74 to 0.77. Calibration results showed relatively close agreement between predicted and observed deaths. The 5-year complete follow-up sample reported some overestimation (11.5%), while the 10-year complete follow-up sample displayed more limited overestimation (1.7%).

**Conclusions:**

Validation results suggest that the PREDICT tool remains essentially relevant for contemporary patients with early stage breast cancer.

## Background

PREDICT is an online prognostication and treatment benefit tool for patients with early stage breast cancer.^1^ The PREDICT online tool aims to help inform clinician and patient decisions about adjuvant therapy following breast cancer surgery. Provided with input of a patient’s clinical characteristics, PREDICT provides personalised prognostic information displayed as 5-year and 10-year overall survival estimates, both with and without adjuvant therapies (chemotherapy, hormone therapy and trastuzumab). Results are presented both in textual format using a frequency-based description of risk and graphically in the form of bar charts with percentages labelled (http://www.predict.nhs.uk/). The PREDICT online tool is popular in the United Kingdom and worldwide with 20,000 visits reported in a single month.^2^

The algorithm behind the online tool was derived primarily from data obtained from the Eastern Cancer Registration and Information Centre (ECRIC) registry in the United Kingdom (east of England).Treatment effectiveness estimates are taken from the Early Breast Cancer Trialists’ Collaborative Group (EBCTCG) meta-analyses of clinical trials.^3^ The first online version of the tool was published in 2010^1^ (v1). A series of updates made since the launch have added new prognostic variables and refined the algorithm’s predictions. The first update published in 2012^4^ added HER2 status as a prognostic marker and allowed calculation of trastuzumab treatment benefit estimates (v.1.2). In 20 14, the tumour proliferative marker Ki-67 was added as an optional prognostic variable^5^ (v1.3). The most recent update, in 2017, refined the model by including age at diagnosis in the breast cancer-specific death prediction as well as recoding tumour size and nodal status variables (v2).^2^ The aim of our study is to conduct an independent validation exercise of the most up-to-date version of the PREDICT algorithm available (v2) using real-world outcomes from the Scottish population of women with breast cancer.

The validity of a prognostic model refers to its ability to accurately predict outcomes for patients, both in the sample from which it was derived (internal validity) and in other populations to which it can be applied (external validity). This study is concerned with external validity and addresses this issue through statistical analysis of the two main performance variables, discrimination and calibration.^6^ The reporting of this validation study follows the “transparent reporting of a multivariable prediction model for individual prognosis or diagnosis” (TRIPOD) guideline.^7^

The high-quality routine data available in Scotland over a long time period allow an assessment of external validity that is greater in scope than previous external validation studies of PREDICT.^1,2,4^ Validation is confined to mortality estimates; treatment benefit is not further considered here as this would require different study designs to provide robust causal inference.

## Methods

### Patient data

Patient-level data were transferred into the National Services Scotland National Safe Haven as an extract from the Scottish Cancer Registry (SCR). SCR is a population-based registry that covers all residents of Scotland (population approximately 5.5 million). National Records of Scotland provides notification of deaths for registry records. All records in the registry with a diagnosis of primary invasive breast cancer (ICD-10 (10th revision of the International Statistical Classification of Diseases and Related Health Problems) C50) diagnosed in the period between January 2001 and December 2015 were retrieved for analysis. Vital status was recorded up to 1 February 2017 in the analysis extract. Deaths due to breast cancer were defined in accordance with the ICD-10 coding system for causes of death, recorded either as the underlying cause of death or one of three secondary causes of death on death notifications. In cases for which there were multiple records of primary breast cancer for the same individual patient, records of non-first occurrences of breast cancer were excluded.

Prognostic factors available in the registry extract included: age at diagnosis, number of lymph nodes examined and number positive, tumour size (maximum pathological diameter in mm), tumour histological grade (categorical: 1–3), mode of detection (screen-detected or symptomatic), oestrogen receptor (ER) status and Her2 status. Treatment status was available in relation to chemotherapy use (binary) and hormone therapy use (binary). Records indicated whether treatment was started or not but did not include information about treatment completion.

This validation analysis followed closely the approaches taken in previous validation studies of PREDICT^1,2,8^ in order to allow comparison of the results. Additional sensitivity analyses, further described below, have been conducted based on unique features of the SCR data.

The data displayed a high level of completeness of all of the variables needed as inputs for the PREDICT model with the exception of HER2 status, trastuzumab use and Ki-67 status. Ki-67 status is not recorded in these data because this marker has not been in routine use in Scotland, and therefore all cases were assigned to the “unknown” category for this variable. HER2 status is only recorded from 2009. Cases with missing data for HER2 were assigned the “unknown” category. This includes 100% of cases from before 2009. The PREDICT algorithm handles “unknown” values in these categories by averaging across the available categories weighted by their frequency in the development data. Furthermore, trastuzumab use is not routinely recorded within the SCR, and therefore it was assumed that trastuzumab was used for all cases with a recorded positive HER2 status and chemotherapy use and in no cases with a recorded negative or unknown status, or positive status with no chemotherapy use. Recent Scottish clinical audit data submitted to a national review reported transtuzumab use for eligible HER2-positive cases is greater than 90% in the majority of health boards.^9^

Chemotherapy use is recorded as a binary variable in SCR, and therefore the generation of chemotherapy is unknown. It was assumed to be second generation for all cases in the primary analysis. A sensitivity analysis varied this assumption to instead assume third-generation chemotherapy for cases which are node positive, under 70 years of age and diagnosed during or after 2006.

PREDICT 10-year prognostic index scores were calculated for each individual case based on their recorded risk factor information using the algorithms supplied by the PREDICT authors (version 2). The scores include the probability of death from all causes, probability of death from breast cancer accounting for competing risk, and adjuvant therapy benefit as the percentage point reduction in the probability of all-cause mortality for each adjuvant therapy. Details of the calculation of the prognostic index are available in Candido Dos Reis et al.^2^

Cases were excluded if the patient was male, had advanced cancer (clinical M stage = 1), did not receive surgery or received neoadjuvant therapy (chemotherapy or hormone therapy recorded prior to date of surgery). Two sensitivity analyses were conducted to test sensitivity of calibration results to the selection criteria applied:^1^ adding an exclusion criteria that the number of lymph nodes examined in node-negative cases must be four or more;^2^ exclusion of T4 cases as well as neoadjuvant cases. The additional exclusion criterion in ref.^1^ creates a sample selected in an equivalent manner to that reported in the development and earlier validation studies of the PREDICT model.^1,8^ Data used in earlier studies were collected in a period prior to widespread use of sentinel node biopsy, and therefore compared with the patients included in this dataset a larger number of nodes were examined.

Cases in which neoadjuvant therapy was used were excluded in the primary analysis because neoadjuvant therapy can alter the prognostic variables recorded in registry data (e.g., tumour size may be reduced) and PREDICT is less relevant as a tool for estimating treatment benefit in such circumstances. Neoadjuvant therapy has become more common in recent years, and therefore we believe that this exclusion criterion is important for the validation of PREDICT, although it has not been applied in previous analyses.

### Statistical methods

Discrimination of the PREDICT score as a prognostic index was assessed by calculating the area under the receiver-operator curve (ROC) (AUC) for both 5- and 10-year all-cause mortality, and 5- and 10-year breast cancer-specific mortality. AUC statistics were calculated separately for ER+ and ER− cases. In addition, Harrell’s c-statistic^10^ was calculated in the primary analysis sample. This is a concordance statistic which can be used with right-censored survival data such as those available in this dataset.

Assessment of calibration was made by comparing the predicted outcomes to the observed outcomes in the validation data. This is reported as the total numbers of deaths predicted and total number of deaths observed in the full sample, and also in selected sub-groups (following Wishart et al.^1^). The 5-year and 10-year periods of complete follow-up were considered. In each case, predicted probabilities of mortality were summed across all individuals for whom complete follow-up was available for the specified period. A third analysis considered individual-specific follow-up periods up to the time of censoring. Predicted probabilities of mortality were calculated using the PREDICT algorithm for each individual’s own potential follow-up time in this analysis. Total numbers of deaths were counted for the selected samples within each of the specified follow-up periods. Differences are reported as relative differences, predicted−observed/observed, over 5 years, 10 years and individual potential follow-up times. Absolute differences (% mortality predicted−% mortality observed) are reported for 5-year and 10-year complete follow-up. Results are reported for all deaths, and separately for breast cancer deaths only (Supplementary Appendix). The sub-groups examined are based on univariate groupings on the variables and levels as described in table S1.

A goodness-of-fit test (Hosmer–Lemeshow test with 10 groups) for survival data was calculated.^11^ This test is based on differences in observed and predicted outcomes in deciles of the prognostic score and a Chi-squared test statistic. Note that we would expect this test to reject the null hypothesis of no difference in observed and predicted outcomes across deciles of score. Small differences in mortality rates could achieve statistical significance due to the very large sample size. Calibration by decile of PREDICT score is also presented graphically as a calibration plot.

A major reason to expect worse calibration in this validation sample compared to the original sample is the time periods in which the cohorts were diagnosed. All patients in the original derivation sample were diagnosed between 1999 and 2003, while this validation sample spans a period from 2001 to 2015. To explore the impact of such time trends a sensitivity analysis was conducted repeating the calculation of calibration statistics on subsets of the data including cases diagnosed from 2001–2005, 2006–2010 and 2011–2015, respectively.

To address potential bias from missing prognostic information, multiple imputation analysis was conducted for all missing prognostic factors. Multiple imputation created 10 datasets using chained equations (MICE).^12^ PREDICT scores were calculated for observations in all imputed datasets. The calibration analysis was repeated with the imputed datasets and the expected and observed deaths were calculated with combination of results across imputed datasets according to Rubin’s rules.^13^

## Results

### Sample selection

**Fig. 1:**
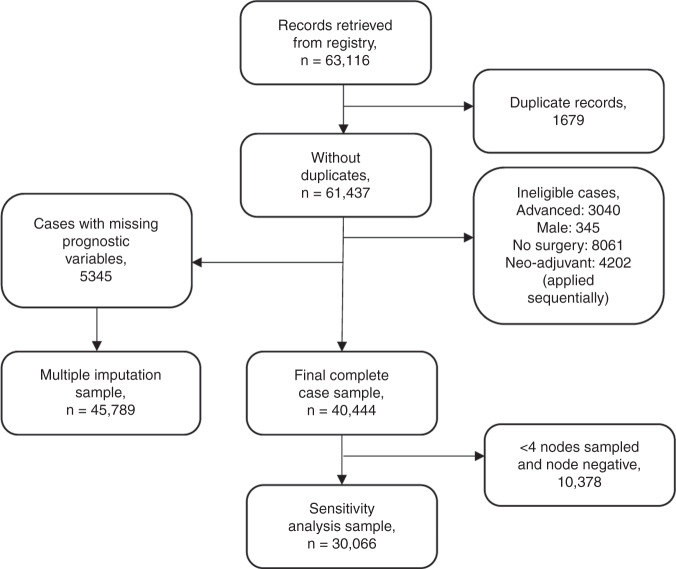
Sample selection flow diagram

A total of 63,116 records were retrieved from the registry. Following removal of “duplicate” records (see Supplementary Appendix for details) and application of the exclusion criteria, a total of 45,789 cases (72.5%) remained in the primary analysis using multiple imputation of missing data. The process is detailed in Fig. 1. The final sample size of complete cases was 40,444. 12% of otherwise eligible cases contained missing prognostic variable data. In most cases only a single variable was missing.

The characteristics of the complete case sample are described in Table 1 alongside the same reported descriptive statistics of the ECRIC cohort.^1^ Notable differences between ECRIC and SCR samples were the somewhat older age distribution, slightly higher use of adjuvant chemotherapy and a higher proportion of screen-detected cases for SCR. There was also a lower proportion of deaths attributed to breast cancer in the SCR data. This may be partly explained by a higher all-cause mortality rate in the Scottish population compared to the East of England population. The age-standardised mortality rate for females was 1025.5 in Scotland compared to 815.6 in the East of England in 2015.^14^

**Table 1:** Characteristics of derivation and validation samples, ECRIC and SCR

	ECRIC 1999–2003 (derivation sample)		SCR 2001–2015 (validation sample)	
Total number of participants	5694		40,444	
Total time at risk (years)	31, 904		285,020	
Median follow-up (years)	5.65		6.41	
Number of breast cancer deaths	737		4922	
Number of other deaths	338		3434	
Annual breast cancer mortality rate	0.023		0.017	
Five-year breast cancer survival rate	0.89		0.91	
Median age at diagnosis, years	58		61	
	Number	(%)	Number	(%)
Age, years				
<35	111	2	506	1.3
35 to 49	1172	21	7094	17.5
50 to 64	2630	46	17054	42.2
65 to 74	1124	20	9975	24.7
75+	657	12	5815	14.4
Nodal status				
0	3532	62	26718	66.1
1	741	13	5824	14.4
2 to 4	806	14	4513	11.2
5 to 9	380	7	1659	4.1
10+	235	5	1484	3.7
Tumour size, mm				
<10	625	11	5542	13.7
10 to 19	2310	41	16,057	39.7
20 to 29	1627	29	10,888	26.9
30 to 49	845	15	6051	15
50+	287	5	1906	4.7
Grade				
I	1005	18	5987	14.8
II	2927	51	19,412	48
III	1762	31	1,4835	36.7
Oestrogen receptor (ER) status				
ER negative	991	17	6311	15.6
ER positive	4703	83	34,133	84.4
Adjuvant therapy				
Chemotherapy	1905	33	14,589	36.1
Endocrine therapy	4268	75	30,252	74.8
Combined chemoendocrine	1122	20	8875	21.9
Screen detected				
Yes	1621	28	15,124	37.4
No	4073	72	25,203	62.3

Source of ECRIC data^1^

### Discrimination

**Fig. 2:**
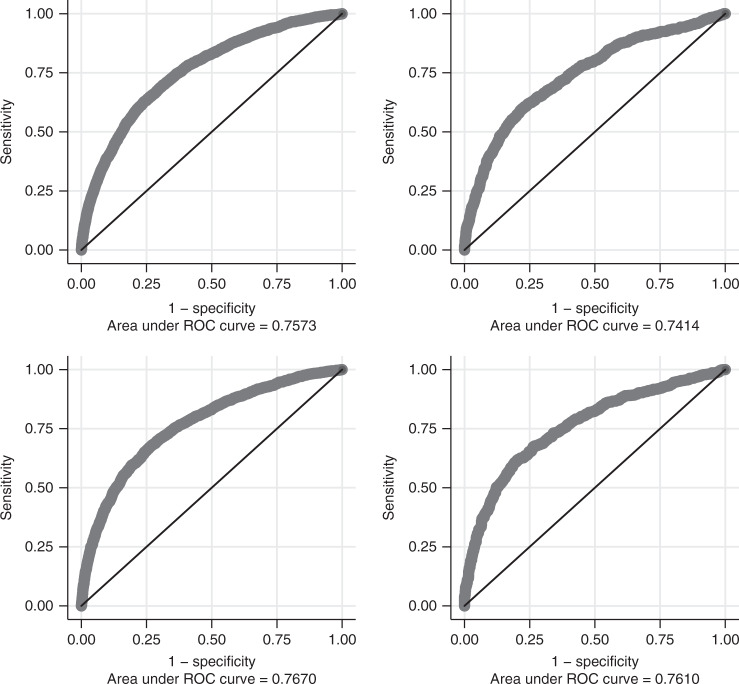
ROC curves, ER+ (left) and ER− (right) cases at 5-year (top) and 10-year (bottom) follow-up. ROC receiver-operator curve

Across ER-positive and ER-negative cases, AUC statistics ranged from 0.75 to 0.78. Performance was similar to that reported in the original data and previous validation exercise (0.76–0.78).^2^ The associated Harrell’s c-statistics were 0.759 for ER positive at 5 years, 0.738 for ER negative at 5 years, 0.749 for ER positive at 10 years and 0.730 for ER negative at 10 years, respectively. AUC statistics for alternative sample selections and outcomes considered across all sensitivity analyses are shown in table S2. ROC curves and the associated AUC statistics are displayed in Fig. 2 for the complete case analysis.

### Calibration

Calibration assessed the accuracy of the probability estimates across specific univariate groupings of individual cases. Total number of predicted and observed deaths in the full sample and in sub-groups according to the defined variables and levels are reported in Table 2.

**Table 2:** Calibration—predicted and observed deaths, full sample and sub-groups

Follow-up	5-Year					10-Year				
Group	*N*	A	P	Relative Mort. Diff. (%)	Absolute Mort. Diff. (% point)	*N*	A	P	Relative Mort. Diff. (%)	Absolute Mort. Diff. (% point)
Total	32,357	4684	4939	5.44	0.79	17,106	5260	5157	−1.96	−0.6
Age <35	426	70	74	5.47	0.94	249	71	79	11.77	3.21
35–49	5851	547	664	21.35	2	3229	621	711	14.44	2.79
50–64	13,700	1246	1418	13.83	1.26	7227	1468	1514	3.11	0.64
65–74	7645	1211	1220	0.71	0.12	3805	1366	1291	−5.5	−1.97
≥75	4735	1610	1563	−2.91	−0.99	2596	1734	1562	−9.9	−6.63
Nodes=0	20,450	2004	2168	8.18	0.8	10,377	2419	2342	−3.22	−0.75
1	4798	692	712	2.83	0.41	2564	783	765	−2.25	−0.7
2–4	4131	886	874	−1.38	−0.3	2414	999	960	−3.9	−1.62
5–9	1492	471	480	1.95	0.6	887	491	476	−3.09	−1.69
10+	1271	559	627	12.02	5.32	724	486	532	9.43	6.32
Tumour size <10	4552	304	338	11.17	0.75	2284	397	371	−6.41	−1.12
10–19	12,289	1155	1280	10.75	1.01	6240	1410	1397	−0.88	−0.21
20–29	8779	1422	1431	0.67	0.11	4736	1654	1532	−7.39	−2.58
30–49	5161	1268	1279	0.88	0.22	2983	1313	1329	1.26	0.54
≥50	1577	535	611	14.17	4.81	863	486	527	8.31	4.71
Grade I	4948	364	335	−7.79	−0.58	2796	554	468	−15.54	−3.07
II	15,058	1680	1801	7.16	0.8	7727	2160	2056	−4.84	−1.35
III	12,351	2640	2803	6.16	1.32	6583	2546	2634	3.43	1.33
Screen	21,059	3909	3929	0.52	0.09	11,919	4349	4165	−4.24	−1.55
Sympt.	11,298	775	1009	30.26	2.07	5187	911	992	8.94	1.57
ER−	5450	1437	1675	16.6	4.37	3037	1237	1307	5.62	2.29
ER+	26,907	3247	3263	0.5	0.06	14,069	4023	3850	−4.29	−1.23

*A* actual deaths, *P* predicted deaths, *Relative Mort. Diff,* difference between actual and predicted as percentage of actual, *Absolute* *Mort. Diff*. difference in 5/10-year mortality (% point)

The 5-year complete follow-up sample show a general pattern of some overestimation of mortality. Overall expected mortality was 5.44% higher relative to observed mortality. In contrast. the 10-year complete follow-up sample showed a small degree of underestimation (−1.96%). In absolute terms, the predicted mortality was 0.79% above observed over 5 years and −0.6% lower over 10 years. In the full sample, using all lengths of follow-up (Table S3), calibration results showed a slightly larger degree of overestimation compared to the 5-year complete follow-up (11.1%).

The degree of overestimation varied between groups, usually between 10 and 25%. Expected mortality was less than observed for the over 75 year age group and grade I cases. Calibration was relatively poor in the group of patients with very large tumour size (>50 mm), cases with very large numbers of nodes involved (10+) and for younger age groups (<35, 35–49, 50–64 years).

**Fig. 3:**
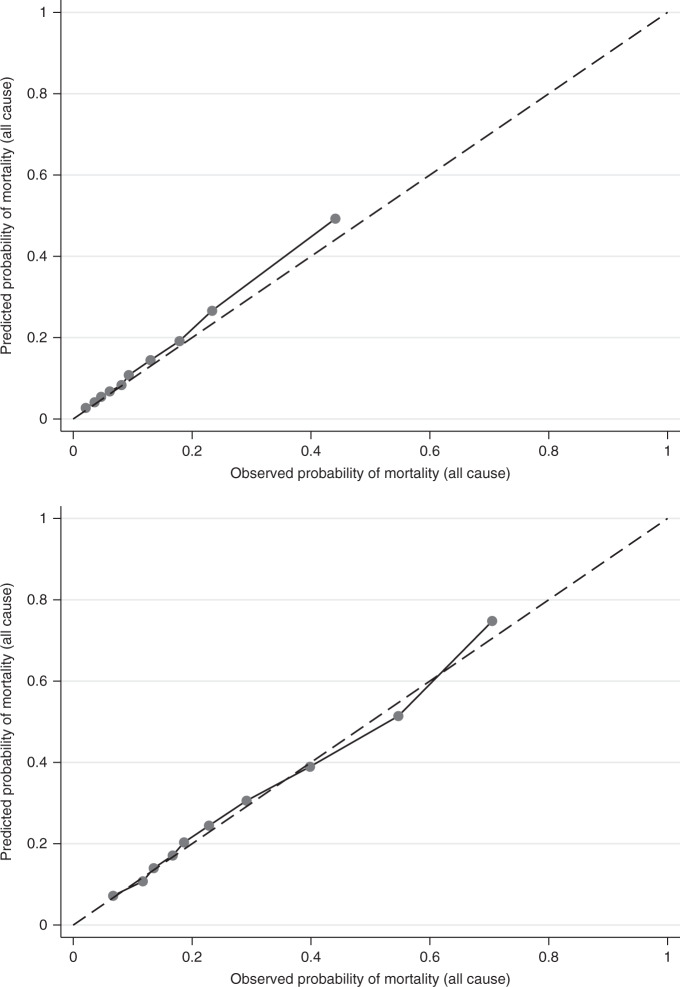
Calibration plots—deciles of PREDICT score, 5-year (top) and 10-year (bottom) all-cause mortality

Predicted and observed numbers of deaths were also calculated by decile of PREDICT score. The results are displayed as a calibration plot in Fig. 3 (complete case only). The Hosmer–Lemeshow test statistic was 49.951 (*P* < 0.001) for 5-year follow-up and 13.449 (*P* = 0.1433) for 10-year follow-up respectively. The figure shows that calibration was very good for lower deciles of PREDICT score and less good for the highest deciles. Calibration across all deciles was good for cases with 10 years of complete follow-up.

Calibration results from the sensitivity analyses are presented in the Supplementary Appendix. The results using complete case data (Table S4) showed slightly worse calibration compared to the primary analysis using multiple imputation. Results were relatively insensitive to using alternative chemotherapy assumptions (Table S5), exclusion of node-negative cases with less than four nodes sampled (Table S6) or exclusion of T4 cases (Table S7). Calibration in relation to breast cancer-specific mortality appeared to be superior to calibration than for all-cause mortality (Table S8). For breast cancer-specific mortality, total predicted mortality was only 2% above observed mortality over 5-year complete follow-up and 4.7% below observed mortality over 10-year complete follow-up. Calibration was relatively poor across sub-groups of age and grade and relatively good across other sub-groups of other variables.

The sensitivity analysis assessing calibration of PREDICT in cohorts diagnosed in the time periods 2001–2005, 2006–2010 and 2011–2015 displayed an apparent time trend in survival outcomes (Table S9-S11). In the 2001–2005 cohort predicted mortality was slightly lower than observed mortality, while in the 2006–2010 and 2011–2015 cohorts the predicted mortality was higher than the observed. The 5-year mortality was underestimated by 6.9% in the first cohort, then overestimated by 17.3% and 33.4% in the two subsequent cohorts. Likewise, in relation to breast cancer-specific mortality (Table S12-S14) there was underestimation of 12% in the first cohort and overestimation of 14.9% and 22.4% in the second and third cohorts.

## Conclusions

In this validation exercise of the PREDICT prognostication tool using an external dataset good performance was demonstrated with regards to both discrimination and calibration, comparable to that reported in the derivation and previous validation data.

The key strength of this validation study is the suitability of the data in terms of both quality and quantity for addressing the research question. The large sample size drawn from the population-based SCR, which is eight times the previous validation samples combined, improves the precision of the results considerably and also allows an assessment of the generalisability of the model to the full population in whom it may be applied. The dataset includes cases diagnosed from 2001 to 2015, allowing its performance in more recent cases to be studied. All cases were followed-up until 2017 and therefore cases diagnosed earlier in the period have a long duration of follow-up.

There are some limitations to this study as a validation exercise. No data were available for so me input parameters: Her2 (before 2009), trastuzumab use, Ki-67 status and generation of chemotherapy used. The PREDICT algorithm allows for unknown Ki-67 or Her2 status. Generation of chemotherapy and trastuzumab use were based on assumptions. Alternative assumptions were explored in a sensitivity analysis which suggests these assumptions were not critical in influencing the results. This analysis cannot assess calibration with regards to Ki-67 status or the potential benefits of including this variable in relation to discrimination. A limitation of the PREDICT model is that is not suitable for providing prognostic estimates in neoadjuvant-treated patients. Exclusion of these cases from the validation sample affects the composition of sample compared to the derivation sample because neoadjuvant-treated patients are predominantly those with moderately poor prognosis. This may improve or worsen model performance depending on if the model performs relatively better or worse for these particular patients.

Calibration was more accurate for some sub-groups than others. In particular, calibration was relatively poor for younger women (35–49, 50–64 years) and relatively good for older women. This is consistent with the results of a validation study performed in a cohort of women aged 65 years or older in the Netherlands.^15^ Some overestimation for 10-year follow-up was observed in the Netherlands cohort, while some underestimation was observed in this cohort. These differences may largely reflect differences in age-specific all-cause mortality rates between these settings; Scotland, the Netherlands and the East of England.

Missing data on individual prognostic variables create some potential for bias. This has been partially addressed by using multiple imputation. However, this relies upon assumptions regarding the pattern of missingness, assumed to be random conditional on observed covariates, and appropriateness of the imputation model. The proportion of cases with any amount of missing data was 12%, which is relatively modest.

The validation results presented in this analysis provides greater confidence in the accuracy of the information given by the PREDICT online tool compared to previous validation studies. Calibration in the overall population appears to be sufficient for decision making purposes. In the Scottish context, version 2 of PREDICT is suitable for providing prognosis and treatment benefit estimates for patients with early breast cancer.

There is some evidence of predictions overestimating mortality for contemporary patients. It should be noted that each percentage point overestimation of absolute mortality risk will result in a fraction of a percentage point reduction in adjuvant chemotherapy benefit estimates (approximately 0 to 1/3 of a percentage point reduction, depending on whether breast cancer mortality and/or other causes of mortality are overestimated). Exploratory results suggest that predictions may become less well calibrated for the most recently diagnosed cohorts (an example of “calibration drift”^6^). This issue is common across prognostic models and is likely to be of particular relevance in early breast cancer because of the continuing introduction of new interventions. If clinically relevant, this could potentially be addressed by an update of the PREDICT model—a version 3—with more recent data used to derive the algorithm’s parameters. Researchers developing and validating prognostic models must strike a balance between using data with longer follow-up, necessarily from cohorts diagnosed further in the past, and using the most recent data, for which follow-up will consequently be shorter. The correct balance will need careful consideration in any future prognostic models in early breast cancer given the potential for calibration drift in this setting.

A limitation of validation studies is that they do not provide a full investigation of the clinical utility or cost effectiveness of using the prognostic model, however accurate it may be. A decision analysis or economic evaluation of use of prognostic models in this setting should therefore be a research priority. This could help to clarify the clinical and cost effectiveness of existing and future alternative prognostic models as well as the addition of new information such as genomic data.

Validation of prognostic models is critical for both providing the necessary evidence for adoption into clinical practice^6^ and for driving continuing improvement in prognostic information. This study provides large-scale investigation of validity of the PREDICT prognostic model for early breast cancer. The results of this investigation suggest that it remains essentially relevant for contemporary patients being diagnosed and managed with invasive breast cancer.

## Electronic supplementary material

Supplementary Appendix

## **Data availability:**

The data used in this study can be accessed through application to electronic Data Research and Innovation Service (eDRIS), a part of the Information Services Division of NHS Scotland. Approval from the Public Benefit and Privacy Panel for Health and Social Care is a requirement for data access.
