# Supplementary material for: Independent validation of the PREDICT breast cancer prognosis prediction tool in 45,789 patients using Scottish Cancer Registry data
Source: Br J Cancer. 2018 Sep 17;119(7):808–14. doi: 10.1038/s41416-018-0256-x (PMC6189179; doi:10.1038/s41416-018-0256-x)
Supplement: Supplementary file 1 — Supplementary Appendix [file 41416_2018_256_MOESM1_ESM.docx]

# Independent validation of the PREDICT breast cancer prognosis prediction tool in 40,444 patients using Scottish Cancer Registry data - Supplementary Appendix

# Tables

## Table S1 - Sub-group categories for calibration reporting

| Variables | Levels |
| --- | --- |
| Age group | 20-34,35-49,50-64,65-74,75+ |
| Nodal status | 0,1,2-4,5-9,10+ |
| Tumour size | <10, 10-19, 20-29, 30-49, 50+ |
| Tumour grade | 1,2,3 |
| ER status | Positive, negative |
| Adjuvant therapy | Chemotherapy, Endocrine therapy, Combined chemoendocrine therapy |
| Screen detected | Yes, No |

## Table S2 - AUC statistics - all sensitivity analyses

| Sample selection | Outcome | 5-year AUC  (95% CI: lb, ub) | | 10-year AUC  (95% CI: lb, ub) | |
| --- | --- | --- | --- | --- | --- |
|  |  | ER+ | ER- | ER+ | ER- |
| Primary analysis –  Multiple Imputation  Neoadjuvant excluded | All-cause mortality | 0.778 (0.76, 0.778) | 0.754 (0.739, 0.769) | 0.775 (0.766, 0.784) | 0.772 (0.755, 0.79) |
| Complete case sample – Neoadjuvant excluded | All-cause mortality | 0.757 (0.748, 0.767) | 0.741 (0.725, 0.758) | 0.767 (0.757, 0.777) | 0.761 (0.742, 0.78) |
| Complete case sample–  Neoadjuvant excluded | Breast cancer specific mortality | 0.774 (0.762, 0.786) | 0.738 (0.721, 0.756) | 0.754 (0.741, 0.766) | 0.727 (0.705, 0.749) |
| Derivation sample criteria –  Complete case  <4 nodes examined, node negative cases excluded  Neoadjuvant included | All-cause mortality | 0.755 (0.745, 0.766) | 0.743 (0.726, 0.76) | 0.767 (0.757, 0.777) | 0.759 (0.739, 0.778) |
| T4 cases excluded  Complete case  Neoadjuvant excluded | All-cause mortality | 0.75 (0.741, 0.76) | 0.736 (0.719, 0.752) | 0.761 (0.751, 0.771) | 0.751 (0.732, 0.77) |
| Alternative chemotherapy assumption –  Complete case  Neoadjuvant excluded | All-cause mortality | 0.757 (0.747, 0.767) | 0.74 (0.723, 0.757) | 0.767 (0.758, 0.777) | 0.762 (0.742, 0.781) |

## Table S3: All individuals evaluated at maximum potential follow-up

| Group | N | A | P | Mort. Diff. (%) |
| --- | --- | --- | --- | --- |
| Total | 40444 | 8356 | 9285 | 11.12 |
| Age <35 | 506 | 112 | 134 | 19.38 |
| 35-49 | 7094 | 934 | 1241 | 32.88 |
| 50-64 | 17054 | 2319 | 2808 | 21.08 |
| 65-74 | 9975 | 2292 | 2445 | 6.66 |
| >=75 | 5815 | 2699 | 2658 | -1.52 |
| Nodes = 0 | 26718 | 4286 | 4599 | 7.31 |
| 1 | 5824 | 1182 | 1324 | 12.03 |
| 2-4 | 4513 | 1304 | 1482 | 13.67 |
| 5-9 | 1659 | 705 | 797 | 13.09 |
| 10+ | 1484 | 766 | 952 | 24.22 |
| Tumour size <10 | 5542 | 531 | 638 | 20.08 |
| 10-19 | 16057 | 2452 | 2734 | 11.5 |
| 20-29 | 10888 | 2642 | 2823 | 6.87 |
| 30-49 | 6051 | 2020 | 2197 | 8.74 |
| >=50 | 1906 | 711 | 894 | 25.73 |
| Grade I | 5987 | 832 | 789 | -5.21 |
| II | 19412 | 3449 | 3775 | 9.46 |
| III | 14835 | 3994 | 4660 | 16.67 |
| Screen | 25203 | 6580 | 7059 | 7.28 |
| Sympt. | 15124 | 1723 | 2171 | 25.99 |
| ER- | 6311 | 1989 | 2312 | 16.24 |
| ER+ | 34133 | 6367 | 6973 | 9.53 |

## Table S4: Complete case analysis

|  | 5-year |  |  |  | 10-year |  |  |  |
| --- | --- | --- | --- | --- | --- | --- | --- | --- |
| Group | N | A | P | Mort. Diff. (%) | N | A | P | Mort. Diff. (%) |
| Total | 28487 | 3848 | 4292 | 11.53 | 14069 | 4125 | 4193 | 1.65 |
| Age <35 | 377 | 62 | 69 | 10.74 | 213 | 62 | 72 | 15.8 |
| 35-49 | 5183 | 466 | 598 | 28.25 | 2675 | 500 | 601 | 20.19 |
| 50-64 | 12169 | 1073 | 1265 | 17.89 | 6012 | 1181 | 1267 | 7.29 |
| 65-74 | 6818 | 1023 | 1085 | 6.08 | 3176 | 1094 | 1076 | -1.63 |
| >=75 | 3940 | 1224 | 1275 | 4.19 | 1993 | 1288 | 1177 | -8.61 |
| Nodes = 0 | 18352 | 1752 | 1936 | 10.5 | 8779 | 2000 | 1976 | -1.21 |
| 1 | 4064 | 521 | 589 | 12.96 | 1994 | 558 | 586 | 5 |
| 2-4 | 3417 | 635 | 698 | 10 | 1837 | 694 | 714 | 2.9 |
| 5-9 | 1300 | 388 | 422 | 8.86 | 717 | 390 | 388 | -0.43 |
| 10+ | 1165 | 491 | 576 | 17.25 | 630 | 418 | 462 | 10.64 |
| Tumour size <10 | 3638 | 207 | 254 | 22.71 | 1648 | 246 | 252 | 2.25 |
| 10-19 | 11168 | 969 | 1145 | 18.16 | 5394 | 1153 | 1196 | 3.74 |
| 20-29 | 7934 | 1204 | 1286 | 6.84 | 4025 | 1360 | 1295 | -4.77 |
| 30-49 | 4432 | 1055 | 1105 | 4.77 | 2360 | 1019 | 1063 | 4.36 |
| >=50 | 1315 | 413 | 501 | 21.34 | 642 | 347 | 387 | 11.52 |
| Grade I | 4299 | 287 | 279 | -2.66 | 2308 | 431 | 372 | -13.67 |
| II | 13322 | 1328 | 1573 | 18.46 | 6357 | 1676 | 1679 | 0.18 |
| III | 10672 | 2180 | 2408 | 10.47 | 5250 | 1952 | 2100 | 7.56 |
| Screen | 18149 | 3112 | 3331 | 7.05 | 9580 | 3311 | 3293 | -0.54 |
| Sympt. | 10222 | 696 | 924 | 32.75 | 4377 | 765 | 851 | 11.29 |
| ER- | 4711 | 1205 | 1407 | 16.8 | 2527 | 993 | 1048 | 5.53 |
| ER+ | 23776 | 2643 | 2884 | 9.13 | 11542 | 3132 | 3145 | 0.42 |

## Table S5: Alternative chemotherapy assumptions

|  | 5-year |  |  |  | 10-year |  |  |  |
| --- | --- | --- | --- | --- | --- | --- | --- | --- |
| Group | N | A | P | Mort. Diff. (%) | N | A | P | Mort. Diff. (%) |
| Total | 28487 | 3848 | 4118 | 7 | 14069 | 4125 | 4046 | -1.91 |
| Age <35 | 377 | 62 | 61 | -1.01 | 213 | 62 | 66 | 6 |
| 35-49 | 5183 | 466 | 538 | 15.44 | 2675 | 500 | 548 | 9.54 |
| 50-64 | 12169 | 1073 | 1180 | 9.96 | 6012 | 1181 | 1197 | 1.34 |
| 65-74 | 6818 | 1023 | 1063 | 3.91 | 3176 | 1094 | 1059 | -3.21 |
| >=75 | 3940 | 1224 | 1275 | 4.19 | 1993 | 1288 | 1177 | -8.61 |
| Nodes = 0 | 18352 | 1752 | 1889 | 7.83 | 8779 | 2000 | 1936 | -3.19 |
| 1 | 4064 | 521 | 561 | 7.65 | 1994 | 558 | 561 | 0.49 |
| 2-4 | 3417 | 635 | 659 | 3.71 | 1837 | 694 | 677 | -2.47 |
| 5-9 | 1300 | 388 | 398 | 2.55 | 717 | 390 | 368 | -5.66 |
| 10+ | 1165 | 491 | 544 | 10.81 | 630 | 418 | 442 | 5.66 |
| Tumour size <10 | 3638 | 207 | 250 | 20.92 | 1648 | 246 | 248 | 0.94 |
| 10-19 | 11168 | 969 | 1109 | 14.41 | 5394 | 1153 | 1163 | 0.84 |
| 20-29 | 7934 | 1204 | 1229 | 2.09 | 4025 | 1360 | 1243 | -8.57 |
| 30-49 | 4432 | 1055 | 1053 | -0.23 | 2360 | 1019 | 1020 | 0.06 |
| >=50 | 1315 | 413 | 477 | 15.45 | 642 | 347 | 372 | 7.24 |
| Grade I | 4299 | 287 | 278 | -3.18 | 2308 | 431 | 370 | -14.21 |
| II | 13322 | 1328 | 1534 | 15.51 | 6357 | 1676 | 1639 | -2.18 |
| III | 10672 | 2180 | 2276 | 4.41 | 5250 | 1952 | 1996 | 2.25 |
| Screen | 18149 | 3112 | 3191 | 2.54 | 9580 | 3311 | 3173 | -4.16 |
| Sympt. | 10222 | 696 | 891 | 28.01 | 4377 | 765 | 825 | 7.89 |
| ER- | 4711 | 1205 | 1329 | 10.31 | 2527 | 993 | 993 | 0.01 |
| ER+ | 23776 | 2643 | 2788 | 5.5 | 11542 | 3132 | 3053 | -2.52 |

## Table S65: Selection node negative as previous validation studies

|  | 5-year |  |  |  | 10-year |  |  |  |
| --- | --- | --- | --- | --- | --- | --- | --- | --- |
| Group | N | A | P | Mort. Diff. (%) | N | A | P | Mort. Diff. (%) |
| Total | 24520 | 3524 | 3896 | 10.56 | 13511 | 3974 | 4061 | 2.19 |
| Age <35 | 349 | 59 | 66 | 12.46 | 208 | 62 | 71 | 14.75 |
| 35-49 | 4580 | 435 | 563 | 29.35 | 2588 | 489 | 591 | 20.8 |
| 50-64 | 10373 | 977 | 1146 | 17.31 | 5778 | 1146 | 1235 | 7.73 |
| 65-74 | 5736 | 937 | 966 | 3.14 | 3041 | 1058 | 1041 | -1.56 |
| >=75 | 3482 | 1116 | 1155 | 3.45 | 1896 | 1219 | 1123 | -7.86 |
| Nodes = 0 | 14385 | 1428 | 1540 | 7.86 | 8221 | 1849 | 1844 | -0.28 |
| 1 | 4064 | 521 | 589 | 12.96 | 1994 | 558 | 586 | 5 |
| 2-4 | 3417 | 635 | 698 | 10 | 1837 | 694 | 714 | 2.9 |
| 5-9 | 1300 | 388 | 422 | 8.86 | 717 | 390 | 388 | -0.43 |
| 10+ | 1165 | 491 | 576 | 17.25 | 630 | 418 | 462 | 10.64 |
| Tumour size <10 | 2808 | 172 | 197 | 14.4 | 1524 | 218 | 231 | 6.13 |
| 10-19 | 9277 | 840 | 977 | 16.33 | 5140 | 1103 | 1144 | 3.71 |
| 20-29 | 7015 | 1099 | 1169 | 6.41 | 3890 | 1305 | 1253 | -4.02 |
| 30-49 | 4147 | 1009 | 1060 | 5.08 | 2322 | 1003 | 1050 | 4.66 |
| >=50 | 1273 | 404 | 492 | 21.9 | 635 | 345 | 383 | 11.16 |
| Grade I | 3543 | 253 | 234 | -7.46 | 2190 | 408 | 353 | -13.58 |
| II | 11323 | 1199 | 1394 | 16.22 | 6084 | 1601 | 1617 | 0.99 |
| III | 9473 | 2020 | 2239 | 10.84 | 5087 | 1901 | 2050 | 7.83 |
| Screen | 16143 | 2885 | 3080 | 6.74 | 9246 | 3200 | 3199 | -0.03 |
| Sympt. | 8267 | 600 | 781 | 30.23 | 4159 | 727 | 815 | 12.16 |
| ER- | 4188 | 1124 | 1301 | 15.76 | 2449 | 972 | 1022 | 5.16 |
| ER+ | 20332 | 2400 | 2595 | 8.12 | 11062 | 3002 | 3039 | 1.23 |

## Table S7: T4 cases excluded

|  | 5-year |  |  |  | 10-year |  |  |  |
| --- | --- | --- | --- | --- | --- | --- | --- | --- |
| Group | N | A | P | Mort. Diff. (%) | N | A | P | Mort. Diff. (%) |
| Total | 29282 | 4023 | 4376 | 8.77 | 14293 | 4165 | 4190 | 0.61 |
| Age <35 | 448 | 82 | 83 | 1.49 | 243 | 74 | 82 | 10.54 |
| 35-49 | 5819 | 613 | 690 | 12.59 | 2920 | 592 | 664 | 12.08 |
| 50-64 | 12582 | 1206 | 1350 | 11.96 | 6167 | 1251 | 1314 | 5.07 |
| 65-74 | 6745 | 1010 | 1075 | 6.45 | 3111 | 1063 | 1046 | -1.58 |
| >=75 | 3688 | 1112 | 1177 | 5.84 | 1852 | 1185 | 1084 | -8.5 |
| Nodes = 0 | 18671 | 1783 | 1946 | 9.16 | 8879 | 2014 | 1965 | -2.45 |
| 1 | 4190 | 549 | 592 | 7.83 | 2032 | 559 | 581 | 4.01 |
| 2-4 | 3610 | 682 | 720 | 5.6 | 1877 | 693 | 711 | 2.66 |
| 5-9 | 1400 | 428 | 450 | 5.15 | 762 | 413 | 408 | -1.31 |
| 10+ | 1215 | 517 | 596 | 15.31 | 627 | 417 | 457 | 9.63 |
| Tumour size <10 | 3793 | 235 | 268 | 14.03 | 1698 | 260 | 259 | -0.36 |
| 10-19 | 11419 | 1021 | 1165 | 14.14 | 5493 | 1184 | 1207 | 1.94 |
| 20-29 | 8089 | 1221 | 1296 | 6.12 | 4089 | 1364 | 1297 | -4.94 |
| 30-49 | 4563 | 1088 | 1116 | 2.61 | 2358 | 1004 | 1041 | 3.71 |
| >=50 | 1418 | 458 | 530 | 15.79 | 655 | 353 | 386 | 9.45 |
| Grade I | 4310 | 278 | 275 | -1.03 | 2311 | 420 | 365 | -13.07 |
| II | 13510 | 1339 | 1569 | 17.15 | 6384 | 1658 | 1653 | -0.31 |
| III | 11248 | 2348 | 2499 | 6.44 | 5437 | 2020 | 2129 | 5.41 |
| Screen | 18880 | 3278 | 3409 | 4 | 9785 | 3349 | 3290 | -1.78 |
| Sympt. | 10286 | 705 | 931 | 32.05 | 4396 | 768 | 853 | 11.11 |
| ER- | 5109 | 1338 | 1495 | 11.74 | 2690 | 1070 | 1091 | 1.99 |
| ER+ | 24173 | 2685 | 2881 | 7.28 | 11603 | 3095 | 3099 | 0.13 |

## Table S8: Breast cancer specific deaths only

|  | 5-year |  |  |  | 10-year |  |  |  |
| --- | --- | --- | --- | --- | --- | --- | --- | --- |
| Group | N | A | P | Mort. Diff. (%) | N | A | P | Mort. Diff. (%) |
| Total | 28487 | 2659 | 2714 | 2.06 | 14069 | 2539 | 2420 | -4.69 |
| Age <35 | 377 | 60 | 67 | 11.58 | 213 | 60 | 69 | 14.84 |
| 35-49 | 5183 | 430 | 548 | 27.41 | 2675 | 453 | 530 | 16.98 |
| 50-64 | 12169 | 787 | 952 | 21.02 | 6012 | 812 | 860 | 5.85 |
| 65-74 | 6818 | 661 | 623 | -5.81 | 3176 | 624 | 538 | -13.82 |
| >=75 | 3940 | 721 | 524 | -27.34 | 1993 | 590 | 424 | -28.15 |
| Nodes = 0 | 18352 | 961 | 919 | -4.33 | 8779 | 953 | 833 | -12.6 |
| 1 | 4064 | 365 | 366 | 0.22 | 1994 | 344 | 342 | -0.53 |
| 2-4 | 3417 | 500 | 514 | 2.84 | 1837 | 502 | 493 | -1.87 |
| 5-9 | 1300 | 321 | 343 | 6.99 | 717 | 308 | 300 | -2.74 |
| 10+ | 1165 | 457 | 512 | 11.94 | 630 | 374 | 398 | 6.44 |
| Tumour size <10 | 3638 | 95 | 95 | 0.29 | 1648 | 102 | 79 | -22.35 |
| 10-19 | 11168 | 537 | 581 | 8.14 | 5394 | 569 | 553 | -2.88 |
| 20-29 | 7934 | 839 | 817 | -2.65 | 4025 | 849 | 765 | -9.89 |
| 30-49 | 4432 | 838 | 808 | -3.55 | 2360 | 741 | 722 | -2.57 |
| >=50 | 1315 | 350 | 413 | 17.92 | 642 | 278 | 301 | 8.36 |
| Grade I | 4299 | 103 | 61 | -40.56 | 2308 | 155 | 83 | -46.43 |
| II | 13322 | 772 | 774 | 0.23 | 6357 | 899 | 798 | -11.22 |
| III | 10672 | 1741 | 1859 | 6.78 | 5250 | 1434 | 1517 | 5.79 |
| Screen | 18149 | 2249 | 2181 | -3.01 | 9580 | 2107 | 1969 | -6.53 |
| Sympt. | 10222 | 376 | 502 | 33.55 | 4377 | 396 | 415 | 4.71 |
| ER- | 4711 | 1019 | 1160 | 13.86 | 2527 | 762 | 769 | 0.97 |
| ER+ | 23776 | 1640 | 1554 | -5.27 | 11542 | 1777 | 1651 | -7.11 |

## Table S9: Time period of diagnosis 2001-2005

|  | 5-year |  |  |  | 10-year |  |  |  |
| --- | --- | --- | --- | --- | --- | --- | --- | --- |
| Group | N | A | P | Mort. Diff. (%) | N | A | P | Mort. Diff. (%) |
| Total | 11606 | 1853 | 1725 | -6.92 | 11606 | 3474 | 3348 | -3.64 |
| Age <35 | 185 | 33 | 32 | -1.9 | 185 | 53 | 55 | 4.66 |
| 35-49 | 2235 | 237 | 246 | 3.83 | 2235 | 423 | 462 | 9.12 |
| 50-64 | 5010 | 530 | 496 | -6.32 | 5010 | 1018 | 1000 | -1.73 |
| 65-74 | 2519 | 483 | 421 | -12.91 | 2519 | 909 | 854 | -6.05 |
| >=75 | 1657 | 570 | 529 | -7.15 | 1657 | 1071 | 976 | -8.85 |
| Nodes = 0 | 7184 | 767 | 737 | -3.93 | 7184 | 1674 | 1578 | -5.71 |
| 1 | 1660 | 247 | 231 | -6.3 | 1660 | 479 | 465 | -2.83 |
| 2-4 | 1536 | 342 | 299 | -12.63 | 1536 | 587 | 567 | -3.35 |
| 5-9 | 608 | 212 | 183 | -13.74 | 608 | 333 | 315 | -5.45 |
| 10+ | 527 | 249 | 242 | -2.77 | 527 | 347 | 368 | 5.97 |
| Tumour size <10 | 1332 | 93 | 86 | -7.33 | 1332 | 201 | 197 | -1.8 |
| 10-19 | 4400 | 441 | 444 | 0.66 | 4400 | 960 | 948 | -1.22 |
| 20-29 | 3377 | 598 | 526 | -12.07 | 3377 | 1174 | 1047 | -10.78 |
| 30-49 | 1964 | 518 | 472 | -8.94 | 1964 | 849 | 842 | -0.81 |
| >=50 | 533 | 203 | 197 | -2.84 | 533 | 290 | 312 | 7.73 |
| Grade I | 1970 | 160 | 128 | -20.09 | 1970 | 378 | 314 | -16.83 |
| II | 5190 | 633 | 619 | -2.28 | 5190 | 1399 | 1347 | -3.72 |
| III | 4298 | 1013 | 955 | -5.72 | 4298 | 1632 | 1648 | 0.96 |
| Screen | 8063 | 1552 | 1419 | -8.55 | 8063 | 2832 | 2682 | -5.28 |
| Sympt. | 3468 | 271 | 282 | 4.19 | 3468 | 607 | 633 | 4.35 |
| ER- | 2104 | 588 | 585 | -0.5 | 2104 | 832 | 822 | -1.18 |
| ER+ | 9502 | 1265 | 1140 | -9.9 | 9502 | 2642 | 2525 | -4.41 |

## Table S10: Time period of diagnosis 2006-2010

|  | 5-year |  |  |  | 10-year |  |  |  |
| --- | --- | --- | --- | --- | --- | --- | --- | --- |
| Group | N | A | P | Mort. Diff. (%) | N | A | P | Mort. Diff. (%) |
| Total | 13571 | 1671 | 1961 | 17.33 | 2463 | 651 | 699 | 7.31 |
| Age <35 | 160 | 26 | 25 | -3.47 | 28 | 9 | 10 | 13.89 |
| 35-49 | 2415 | 189 | 244 | 29.22 | 440 | 77 | 86 | 11.83 |
| 50-64 | 5737 | 466 | 564 | 20.99 | 1002 | 163 | 196 | 20.52 |
| 65-74 | 3416 | 446 | 522 | 17.15 | 657 | 185 | 205 | 10.72 |
| >=75 | 1843 | 544 | 605 | 11.2 | 336 | 217 | 201 | -7.41 |
| Nodes = 0 | 8882 | 817 | 925 | 13.26 | 1595 | 326 | 358 | 9.73 |
| 1 | 1934 | 222 | 269 | 21.34 | 334 | 79 | 95 | 20.61 |
| 2-4 | 1553 | 251 | 299 | 19.06 | 301 | 107 | 110 | 2.37 |
| 5-9 | 574 | 146 | 178 | 22.01 | 109 | 57 | 53 | -6.9 |
| 10+ | 541 | 212 | 258 | 21.85 | 103 | 71 | 74 | 4.14 |
| Tumour size <10 | 1801 | 96 | 128 | 33.43 | 316 | 45 | 51 | 13.17 |
| 10-19 | 5408 | 441 | 537 | 21.82 | 994 | 193 | 214 | 11.08 |
| 20-29 | 3671 | 501 | 568 | 13.39 | 648 | 186 | 196 | 5.4 |
| 30-49 | 2037 | 450 | 490 | 8.8 | 396 | 170 | 178 | 4.42 |
| >=50 | 654 | 183 | 238 | 29.79 | 109 | 57 | 60 | 4.77 |
| Grade I | 1860 | 103 | 120 | 16.52 | 338 | 53 | 55 | 4.51 |
| II | 6463 | 579 | 743 | 28.3 | 1167 | 277 | 293 | 5.61 |
| III | 5208 | 983 | 1092 | 11.12 | 952 | 320 | 348 | 8.83 |
| Screen | 8268 | 1312 | 1465 | 11.68 | 1517 | 479 | 491 | 2.44 |
| Sympt. | 5262 | 349 | 483 | 38.41 | 909 | 158 | 192 | 21.49 |
| ER- | 2196 | 533 | 629 | 17.96 | 423 | 161 | 171 | 6.17 |
| ER+ | 11375 | 1138 | 1332 | 17.03 | 2040 | 490 | 528 | 7.68 |

## Table S11: Time period of diagnosis 2011-2015

|  |  | 5-year |  |  |  | 10-year |  |  |  |
| --- | --- | --- | --- | --- | --- | --- | --- | --- | --- |
| Group |  | N | A | P | Mort. Diff. (%) | N | A | P | Mort. Diff. (%) |
| Total |  | 3310 | 324 | 432 | 33.39 | n/a | n/a | n/a | n/a |
| Age <35 |  | 32 | 3 | 4 | 30.16 |  |  |  |  |
| 35-49 |  | 533 | 40 | 48 | 19.12 |  |  |  |  |
| 50-64 |  | 1422 | 77 | 120 | 55.29 |  |  |  |  |
| 65-74 |  | 883 | 94 | 120 | 27.59 |  |  |  |  |
| >=75 |  | 440 | 110 | 141 | 28.29 |  |  |  |  |
| Nodes = 0 |  | 2286 | 168 | 227 | 35.13 |  |  |  |  |
| 1 |  | 470 | 52 | 60 | 15.42 |  |  |  |  |
| 2-4 |  | 328 | 42 | 61 | 45.1 |  |  |  |  |
| 5-9 |  | 118 | 30 | 37 | 22.96 |  |  |  |  |
| 10+ |  | 97 | 30 | 44 | 45.43 |  |  |  |  |
| Tumour size <10 |  | 505 | 18 | 36 | 100.12 |  |  |  |  |
| 10-19 |  | 1360 | 87 | 128 | 46.56 |  |  |  |  |
| 20-29 |  | 886 | 105 | 135 | 28.84 |  |  |  |  |
| 30-49 |  | 431 | 87 | 91 | 4.96 |  |  |  |  |
| >=50 |  | 128 | 27 | 42 | 55.76 |  |  |  |  |
| Grade I |  | 469 | 24 | 30 | 25 |  |  |  |  |
| II |  | 1669 | 116 | 173 | 48.82 |  |  |  |  |
| III |  | 1166 | 184 | 229 | 24.32 |  |  |  |  |
| Screen |  | 1818 | 248 | 307 | 23.63 |  |  |  |  |
| Sympt. |  | 1492 | 76 | 126 | 65.22 |  |  |  |  |
| ER- |  | 411 | 84 | 115 | 37.46 |  |  |  |  |
| ER+ |  | 2899 | 240 | 317 | 31.96 |  |  |  |  |

## Table S12: Breast cancer specific 2001-2005

##

|  | 5-year |  |  |  | 10-year |  |  |  |
| --- | --- | --- | --- | --- | --- | --- | --- | --- |
| Group | N | A | P | Mort. Diff. (%) | N | A | P | Mort. Diff. (%) |
| Total | 11606 | 1329 | 1170 | -11.98 | 11606 | 2168 | 2015 | -7.05 |
| Age <35 | 185 | 33 | 35 | 6.38 | 185 | 51 | 58 | 13.46 |
| 35-49 | 2235 | 225 | 252 | 11.84 | 2235 | 382 | 446 | 16.8 |
| 50-64 | 5010 | 405 | 407 | 0.45 | 5010 | 713 | 719 | 0.87 |
| 65-74 | 2519 | 332 | 256 | -22.77 | 2519 | 524 | 440 | -16.05 |
| >=75 | 1657 | 334 | 220 | -34.2 | 1657 | 498 | 352 | -29.3 |
| Nodes = 0 | 7184 | 436 | 365 | -16.36 | 7184 | 803 | 680 | -15.27 |
| 1 | 1660 | 176 | 156 | -11.61 | 1660 | 301 | 285 | -5.25 |
| 2-4 | 1536 | 274 | 233 | -15.05 | 1536 | 435 | 415 | -4.61 |
| 5-9 | 608 | 176 | 157 | -10.75 | 608 | 267 | 256 | -3.95 |
| 10+ | 527 | 234 | 230 | -1.81 | 527 | 314 | 332 | 5.66 |
| Tumour size <10 | 1332 | 55 | 34 | -37.47 | 1332 | 89 | 63 | -29.46 |
| 10-19 | 4400 | 255 | 244 | -4.15 | 4400 | 479 | 455 | -5.03 |
| 20-29 | 3377 | 417 | 356 | -14.75 | 3377 | 741 | 643 | -13.16 |
| 30-49 | 1964 | 428 | 366 | -14.5 | 1964 | 624 | 601 | -3.64 |
| >=50 | 533 | 174 | 169 | -2.59 | 533 | 235 | 253 | 7.55 |
| Grade I | 1970 | 70 | 30 | -57.68 | 1970 | 138 | 71 | -48.39 |
| II | 5190 | 388 | 326 | -16.09 | 5190 | 770 | 661 | -14.13 |
| III | 4298 | 830 | 799 | -3.78 | 4298 | 1210 | 1262 | 4.31 |
| Screen | 8063 | 1148 | 984 | -14.32 | 8063 | 1817 | 1671 | -8.02 |
| Sympt. | 3468 | 156 | 166 | 6.22 | 3468 | 324 | 319 | -1.59 |
| ER- | 2104 | 514 | 516 | 0.47 | 2104 | 642 | 638 | -0.55 |
| ER+ | 9502 | 815 | 653 | -19.84 | 9502 | 1526 | 1377 | -9.78 |

## Table S13: Breast cancer specific 2006-2010

##

|  | 5-year |  |  |  | 10-year |  |  |  |
| --- | --- | --- | --- | --- | --- | --- | --- | --- |
| Group | N | A | P | Mort. Diff. (%) | N | A | P | Mort. Diff. (%) |
| Total | 13571 | 1118 | 1285 | 14.9 | 2463 | 371 | 405 | 9.12 |
| Age <35 | 160 | 24 | 28 | 14.65 | 28 | 9 | 11 | 22.64 |
| 35-49 | 2415 | 171 | 249 | 45.35 | 440 | 71 | 84 | 17.97 |
| 50-64 | 5737 | 326 | 456 | 39.96 | 1002 | 99 | 140 | 41.73 |
| 65-74 | 3416 | 272 | 304 | 11.63 | 657 | 100 | 98 | -2.14 |
| >=75 | 1843 | 325 | 249 | -23.52 | 336 | 92 | 72 | -21.88 |
| Nodes = 0 | 8882 | 435 | 451 | 3.74 | 1595 | 150 | 153 | 1.73 |
| 1 | 1934 | 154 | 174 | 12.92 | 334 | 43 | 57 | 32.54 |
| 2-4 | 1553 | 189 | 235 | 24.25 | 301 | 67 | 78 | 15.94 |
| 5-9 | 574 | 123 | 156 | 26.68 | 109 | 41 | 43 | 5.1 |
| 10+ | 541 | 197 | 242 | 23.08 | 103 | 60 | 66 | 10.56 |
| Tumour size <10 | 1801 | 34 | 49 | 43.45 | 316 | 13 | 16 | 26.28 |
| 10-19 | 5408 | 236 | 278 | 17.69 | 994 | 90 | 98 | 8.55 |
| 20-29 | 3671 | 352 | 375 | 6.57 | 648 | 108 | 122 | 12.56 |
| 30-49 | 2037 | 342 | 375 | 9.74 | 396 | 117 | 121 | 3.11 |
| >=50 | 654 | 154 | 208 | 34.81 | 109 | 43 | 49 | 12.83 |
| Grade I | 1860 | 29 | 26 | -10.56 | 338 | 17 | 12 | -30.52 |
| II | 6463 | 321 | 372 | 15.98 | 1167 | 129 | 137 | 6.15 |
| III | 5208 | 766 | 883 | 15.24 | 952 | 224 | 255 | 13.81 |
| Screen | 8268 | 933 | 1001 | 7.34 | 1517 | 290 | 298 | 2.79 |
| Sympt. | 5262 | 176 | 273 | 55.26 | 909 | 72 | 96 | 33.04 |
| ER- | 2196 | 440 | 547 | 24.22 | 423 | 120 | 131 | 9.13 |
| ER+ | 11375 | 678 | 738 | 8.85 | 2040 | 251 | 274 | 9.11 |

## Table S14: Breast cancer specific 2011-2015

##

|  | 5-year |  |  |  | 10-year |  |  |  |
| --- | --- | --- | --- | --- | --- | --- | --- | --- |
| Group | N | A | P | Mort. Diff. (%) | N | A | P | Mort. Diff. (%) |
| Total | 3310 | 212 | 259 | 22.38 | n/a | n/a | n/a | n/a |
| Age <35 | 32 | 3 | 4 | 44.25 |  |  |  |  |
| 35-49 | 533 | 34 | 48 | 40.3 |  |  |  |  |
| 50-64 | 1422 | 56 | 89 | 59.5 |  |  |  |  |
| 65-74 | 883 | 57 | 63 | 9.72 |  |  |  |  |
| >=75 | 440 | 62 | 56 | -10.4 |  |  |  |  |
| Nodes = 0 | 2286 | 90 | 103 | 14.94 |  |  |  |  |
| 1 | 470 | 35 | 36 | 3.84 |  |  |  |  |
| 2-4 | 328 | 37 | 47 | 25.93 |  |  |  |  |
| 5-9 | 118 | 22 | 31 | 38.89 |  |  |  |  |
| 10+ | 97 | 26 | 39 | 51.29 |  |  |  |  |
| Tumour size <10 | 505 | 6 | 12 | 101.87 |  |  |  |  |
| 10-19 | 1360 | 46 | 59 | 27.28 |  |  |  |  |
| 20-29 | 886 | 70 | 86 | 23.05 |  |  |  |  |
| 30-49 | 431 | 68 | 67 | -1.43 |  |  |  |  |
| >=50 | 128 | 22 | 36 | 61.87 |  |  |  |  |
| Grade I | 469 | 4 | 6 | 41.55 |  |  |  |  |
| II | 1669 | 63 | 76 | 20.51 |  |  |  |  |
| III | 1166 | 145 | 178 | 22.46 |  |  |  |  |
| Screen | 1818 | 168 | 196 | 16.82 |  |  |  |  |
| Sympt. | 1492 | 44 | 63 | 43.61 |  |  |  |  |
| ER- | 411 | 65 | 97 | 49.53 |  |  |  |  |
| ER+ | 2899 | 147 | 162 | 10.37 |  |  |  |  |

# Duplicate record removal process

For persons (unique CHI numbers) with more than one primary breast cancer record the process used to select one record from among the duplicates was as follows:

1. If all variables available match exactly, the first of the identical records was selected
2. If all prognostic and outcomes variables used in the analysis match exactly the first (chronologically) record was selected.
3. If any prognostic and outcomes variables do not match and records fall within 6 months of each other, the record with worst prognosis – determined by the worst PREDICT score – was selected.
4. If any prognostic and outcomes variables do not match and records fall out with 6 months of each other, the first record in chronological order was selected.

Note that duplicate records may relate to individuals with multiple primary tumours, in which case we seek to select only the first primary tumour. In cases where there may have synchronous multiple primary tumours we take the tumour with the worse prognosis. Note that synchronous cases may also reflect recording error however we believe this to be minor in this cancer registry dataset.

**Note on multiple imputation procedure:**

The imputation models were specified as logistic regression for ER status, method of detection, chemotherapy use and hormone therapy use, ordered logit regression for grade, Poisson regression for number of positive nodes and truncated regression for tumour size. Predictor variables in each equation included age (present for all observations), mortality and time-to-mortality outcomes, and the remaining prognostic variables.

This analysis addresses some potential bias arising from missing data on prognostic variables. It assumes that the missing data is random in relation to the true values of the missing variables conditional on the values of the other prognostic variables and outcomes. For example, if tumour size is more likely to be missing in cases with high numbers of nodes involved, and these cases have systematically worse outcome, then this potentially would bias the complete case analysis. Multiple imputation will allow inclusion of these cases in the analysis and remove the potential bias under the assumption about the nature of the missing data being met. The missing tumour size variable is (multiply) imputed such that in each case the expected value is conditional on the observed level of the other prognostic variables. If the missing tumour size cases had true tumour size values that were systematically different to the conditional expected values then this would violate the missing at random assumption and there will still be potential for bias due to the missing data.
